# Women and Violence: Alexithymia, Relational Competence and Styles, and Satisfaction with Life: A Comparative Profile Analysis

**DOI:** 10.3390/bs11110147

**Published:** 2021-10-26

**Authors:** Stefania Mannarini, Federica Taccini, Alessandro Alberto Rossi

**Affiliations:** 1 Section of Applied Psychology, Department of Philosophy, Sociology, Education and Applied Psychology, University of Padova, 35131 Padova, Italy; stefania.mannarini@unipd.it (S.M.); federica.taccini@phd.unipd.it (F.T.); 2 Interdepartmental Center for Family Research, University of Padova, 35131 Padova, Italy

**Keywords:** interpersonal violence, offenders, victims, women, profile analysis, clinical psychology, dynamic psychology

## Abstract

**Background:** This research investigates the two sides of violence by profiling female interpersonal violence offenders (*IVO*) and female interpersonal violence victims (*IVV*). These groups of women have been compared on three key variables within the context of violence: satisfaction with life (SWL), alexithymia, and relational styles—defined according to the Relational Competence Theory (RCT). Regarding the latter, the experience of functional or dysfunctional relational styles in childhood and adult relationships has been evaluated and compared with both groups of women. **Methods:** This study involved 131 women: *IVO* (*n* = 41; enrolled in a penitentiary), *IVV* (*n* = 41; enrolled in an anti-violence center), and a control group (*CG*; *n* = 49; enrolled from the general population). Profile analysis was performed. **Results:** Female *IVO* showed low SWL, high levels of alexithymia, and a pattern of mixed relational styles with both parents and the current partner. Female *IVV* revealed low SWL, low levels of alexithymia and dysfunctional relational styles with both parents and current partner. Women from the *CG* showed high SWL, absence of alexithymia and functional relational styles with both parents and current partner. **Conclusions:** The profiles outlined in this study are extremely informative regarding alexithymia, relational styles, and SWL in both female *IVV* and *IVO*. Clinical interventions for both groups of women should be developed relying on these results.

## 1. Introduction

The diffusion of interpersonal violence (IV) represents a serious social emergency with a strong impact on the psychological and emotional health of the people involved [1,2,3]. IV refers to the violence that takes place among people, and it includes family and community violence and intimate partner violence (IPV) [4]. In recent years, several studies have highlighted that IV is a constantly growing dramatic phenomenon that affects both men and females: on the one hand, women may be significantly more likely to be victims of physical, sexual, and psychological violence, such as control and power abuse [5,6]. On the other hand, several studies highlighted the active role of women in carrying out IV, such as verbal, emotional, and psychological abuse, sometimes reaching physical violence [5,7].

Regardless of the role of the victim or the offender, some characteristic patterns seem to be recurrent in these two categories in relation to specific psychological variables. Indeed, as a consequence of the negative experiences of IV, victims are more likely to experience anxiety, depression, symptoms of post-traumatic stress disorder, and suicide attempts [8,9,10]. Among violent offenders, depression, stress, guilt, and shame are frequently reported [11,12]. In addition, both victims and offenders reported low satisfaction with their lives [13,14]. Satisfaction with life corresponds to *«individual’s overall experience with life»* [15] and it affects individuals’ academic outcomes, social connectedness, self-efficacy and the actual accomplishment of life’s purposes [15,16]. Furthermore, SWL also affects psychological well-being: indeed, higher SWL appears to be associated with lower mental health problems [16]. Therefore, considering its relevance, more studies should be conducted with the aim of comparing victims and perpetrators in this regard, since only a few have focused their attention on SWL in these populations.

Additionally, alexithymic traits [17,18,19] seem to characterize both IV victims (*IVV*) and IV offenders (*IVO*) [20,21]. Alexithymic traits refer to difficulties in identifying, labeling, cognitive processing, and regulating emotions [17,22]. To date, few studies have explored alexithymic traits within *IVV* and *IVO* and even fewer have focused on women [21,22,23,24]. On the one hand, *IVV* females report higher alexithymic traits and difficulties with affect regulation compared with the general population [19,20,24,25,26]. There is still a debate as to whether alexithymia represents either a risk factor for IPV experience [20,26] or a consequence of the traumatic phenomenon in this population, as victims may experience emotional numbness as a consequence of trauma [27,28]. On the other hand, *IVO* women with alexithymic traits are more at risk for intimate partner violence [21]. Indeed, difficulties in understanding affective states as well as in communicating emotions may lead to relying on maladaptive emotion regulation strategies, which may burst into violent acts to end the negative emotions experienced [21,23,24,26,29].

Moreover, research suggested that alexithymic traits could be associated with previous adverse relational experiences [26], such as dysfunctional relational styles of the family of origin and physical and sexual assault [30,31,32]. Consequently, parents’ relational competence and parenting styles affect children’s ability to identify and manage emotions [30,33]. According to Relational Competence Theory (RCT) [34,35,36], relational competence corresponds to the totality of personality characteristics, interpersonal skills, and relational styles an individual develops interacting with others in different contexts [30,31,32]. Consequently, previous experiences of a violent environment may be one of the factors (e.g., lack of education, low economic level, etc.) that contribute to the passive and/or active reenactment of violent conducts in adult relationships. Indeed, possible trauma in parents’ history may be transmitted in an intergenerational way to the offspring [34,37,38,39,40,41]. Hence, relational competence plays a central role in violent interpersonal dynamics [33,34,35,36]. In this regard, three interpersonal styles were proposed: the abusive-apathetic (AA) style, the reactive-repetitive (RR) one, and the creative-conductive (CC) style [37,38]. The AA style is characterized by behaviors related to apathy, violence, or neglect. Therefore, people with high levels of AA can react to events aggressively or in an unreliable and incompetent way. Therefore, they may not be able to establish intimate relationships with others. People with higher levels of the RR style may react immediately or in a delayed way to events to the point of sometimes withdrawing from situations to keep the interpersonal dynamics unaltered. This may have negative consequences on relationships, suggesting a lack of intimacy with their significant others. Individuals with either higher levels of the RR style or of the AA one seem not to consider future consequences of their actions. On the contrary, people with higher levels of the CC style show creativity and conductivity in interpersonal dynamics. They may remain calm in stressful situations, deal effectively with both positive and negative events, and establish functional interpersonal relationships [37]. Therefore, according to the RCT, interactions with significant others can play an important role in people’s relational competence and styles [37,38].

Despite several studies investigating both female *IVV* and *IVO*, only a few compared these two faces of violence (victims and offenders) and none of them analysed the psychological variables related to IV mentioned above. In addition, several studies attempted to define a specific pattern of characteristics of both *IVV* and *IVO* [39,40,41]. However, research focused specifically on the above-mentioned variables is still lacking. Thus, this study aimed to profile and compare, in an exploratory way, three groups of women: (I) victims of violence, (II) violent offenders, and (III) an age-matched control group. These groups have been compared on alexithymia, relational competence and styles, and satisfaction with life.

## 2. Methods

### 2.1. Procedure and Inclusion/Exclusion Criteria

Each participant was individually recruited, and the research survey was administered individually. Each participant voluntarily agreed to participate in the study and signed a written informed consent. The research project was previously approved by the Ethics Committee of the University of Padua, Italy

Female *IVO* were recruited into a penitentiary in the north of Italy, while female *IVV* were enrolled in a women’s shelter in north of Italy. The research survey was administered within 3 to 15 months after arrival both at the penitentiary and at the women’s shelter. Finally, using the snowball sampling technique, the *CG* was enrolled from the general population in Padua by means of personal invitations, advertising in the University, in cafes, and libraries.

General inclusion criteria consisted of the following: being over 18 years old, being a native Italian speaker, being in a relationship (or having ended up in the relationship at maximum three months before the survey administration). In addition, the general exclusion criteria consisted of the following: inability to complete the assessment procedure due to illiteracy, cognitive, and/or vision impairments.

Specific inclusion criteria were applied for each group. Women should have enacted (*IVO* group only) or suffered (*IVV* group only) at least one episode of interpersonal violence within the last 6–18 months. Contrarily, women in the *CG* should neither have enacted nor have ever been subjected to any kind of (interpersonal) violence.

### 2.2. Sample Size Determination

The minimum sample size required was computed a priori with G*Power software [42]. The Multivariate Analysis of Variance (MANOVA) family of statistics was chosen [43,44,45]. Three independent groups of participants were specified (*IVO* vs. *IVV* vs. *CG*) and measured with 8 different psychological scales (see the section ‘instruments’). According to guidelines [46], a priori statistics were set at small values (small effects)—considering the lack of previous/similar studies from which realistic estimates of effect sizes were derived. In particular, Pillai’s trace (V) was set to 0.2 (effects provide a minimum contribution [47]), resulting in a small effect size: *f^2^*(V) = 0.11 [46,48]. The type I error (α) was set at 0.05 (two-sided) and the Power (1-β) was set at 0.80 [46]. The results showed that there is a more that 81% chance of correctly rejecting the null hypothesis of no significant effect of the interaction with an overall sample of 96 subjects, a minimum of 32 participants per group.

### 2.3. Participants

A total of 142 women were contacted. Of the total of participants, 11 women did not complete the procedure: 9 female *IVO*, 1 female *IVV*, and 1 female *CG*. Thus, the overall group of participants consisted of 131 individuals. More in detail, there were 41 *IVO* women, aged 20 to 63 years (*mean* = 38.80, *SD* = 11.25), 49 *CG* women, aged 18 to 62 years (*mean* = 36.71, *SD* = 10.93), and 41 *IVV* women, aged 21 to 62 years (*mean* = 36.98, *SD* = 11.50).

### 2.4. Instruments

#### 2.4.1. Toronto Alexithymia Scale 20 (TAS-20)

The TAS-20 [17,49] is the most widely used self-report questionnaire for measuring alexithymia. TAS-20 was used with women who enacted violence as well as with female *IVV* [20,21]. The TAS-20 evaluates the three main dimensions of alexithymia [17]: (A) difficulties in identifying feelings; (B) difficulty describing feelings to other people; and (C) externally oriented thinking. However, a general total score is strongly assumed [17,49,50]. It consists of 20 items rated on a 5-point Likert scale (ranging from 1 = “strongly disagree” to 5 = “strongly agree”), with higher scores reflecting higher levels of alexithymia. In this study, the Italian version of TAS-20 was used and provided good internal consistency: Cronbach’s Alpha = 0.831.

#### 2.4.2. Questionnaire of Relational Styles (Questionario Sugli Stili Relazionali—QSR)

The QSR [38] is a self-report questionnaire measuring relational competence styles according to Relational Competence Theory [51]. Moreover, also in this case, QSR was already used in studies with female *IVO* as well as female *IVV* [38]. The QSR consists of two different parts, each composed of three different scales that assess the three main relational styles: (1) Abusive-Apathetic, ‘AA’; (2) Reactive-Repetitive, ‘RR’; and (3) Creative-Conductive, ‘CC’. Thus, six different total scores are provided. Higher scores reflect higher levels of the specific relational style. In this study, the Italian version of the QSR was used.

The first part of the QSR consists of 21 items rated on a 4-point Likert-type scale (ranging from 1 = *“never”* to 4 = *“always”*). It assesses the relational styles experienced with parent(s), thus providing three different scores: (1) Abusive-Apathetic parent(s), ‘pAA’; (2) Reactive-Repetitive parent(s), ‘pRR’; and (3) Creative-Conductive parent(s), ‘pCC’. In this study, each of these three QSR scales provided good internal consistency: ‘pAA’: Cronbach’s alpha = 0.858; ‘pRR’: Cronbach’s alpha = 0.757; ‘pCC’; Cronbach’s alpha = 0.805.

The second part of the QSR is made up of 24 items rated on a 5-point Likert-type scale (ranging from 1 = *“almost never”* to 5 = *“almost always”*). It assesses relational styles related to current relationships (i.e., significant others and/or current family and/or friends) and—even in this case—provides three different scores: (4) Abusive-Apathetic current relationship, ‘cAA’; (5) Reactive-Repetitive current relationship, ‘cRR’; and (6) Creative-Conductive current relationship, ‘cCC’. In this study, each of these three QSR scales provided good internal consistency: ‘cAA’: Cronbach’s alpha = 0.685; ‘cRR’: Cronbach’s alpha = 0.683; ‘cCC’: Cronbach’s alpha = 0.837.

#### 2.4.3. The Satisfaction with Life Scale (SWLS)

SWLS [52,53] is the most widely used self-report questionnaire to measure “satisfaction with his/her own life” in female *IVO* as well as in *IVV* [54]. The SWLS consists of 5 items rated on a 7-point Likert scale (ranging from 1 = *“strongly disagree”* to 7 = *“strongly agree”*). In this case, higher scores reflect greater satisfaction with their own life. In this study, the Italian version of the SWLS was used and it provided good internal consistency: Cronbach’s alpha = 0.833.

### 2.5. Statistical Analyses

Data analysis was performed using R software and the following packages: ‘esvis’ [55], ‘ggplot2′ [56], ‘overlapping’ [57], ‘profileR’ [43,58], and ‘psych’ [59].

First, according to the guidelines [45], both univariate and multivariate normality, linearity, multicollinearity, and homogeneity of covariances matrices were inspected.

Second, a profile analysis (PA) was implemented and performed. PA allows to determine and interpret to what extent the three groups of women (independent variable) revealed different profiles on the variables implied in IV (dependent variables)—quantifying the degree of dissimilarity between profiles [43,58,60,61,62]. PA is a special application of the multivariate analysis of variance (MANOVA) test; thus, it is a multivariate approach to test mean differences towards three specific statistics: (I) *parallelism*, (II) *level* *equality*, and (III) *flatness* [44,45,62,63]. (I) *Parallelism* assesses whether the shape of two profiles is analogous and symmetrical (parallel) between different groups—between-subject general statistic. To assess parallelism, 7 segments were artificially created: (A) Alexithymia vs. pAA; (B) pAA vs. pRR; (C) pRR vs. pCC; (D) pCC vs. cAA; (E) cAA vs. cRR; (F) cRR vs. cCC; (G) cCC vs. satisfaction with life. Each segment represents the slope of the line between the means of two close variables. These slopes are used to test whether the difference between two segments is the same between groups. The overall multivariate effect was assessed using the Wilks’ lambda (Λ). (II) *Level equality* refers to the degree of similarity in means of scores across all dependent variables across all groups—general between-subject statistic. To test level equality, several focused multivariate comparisons between groups were performed. The Games-Howell post hoc test was chosen to evaluate the univariate comparison analysis exploring mean differences between groups [64]. Lastly, (III) *Flatness* aimed to determine whether (within each profile) each variable score yielded a similar response to the following variable: general within-subjects statistic [45]. To test flatness, several focused univariates repeated measures comparisons were also performed for each group to assess within group effects. Furthermore, several focused univariates repeated measures comparisons were also performed for each segment of each group profile. In this case, the Bonferroni post hoc test was chosen to assess univariate comparison analysis exploring mean differences between segments [64].

According to the guidelines, before performing PA, all dependent variables were rescaled to z-scores [43,45].

For multiple comparisons, the strength of differences was interpreted using Cohen’s *f* [46]. For pairwise comparisons, Hedge’s *g* [65] and the ‘overlapping index’ *η* [57] were used to quantify both the strength of differences and the overlap of kernel density distribution, respectively. For repeated measures comparisons, the adapted formula of Cohen’s *d* was used [46]. The following benchmarks [46,57] were used: null (*f* < 0.10; *g*, *η*, *d* < 0.20), small (*f* from 0.10 to 0.25; *g*, *η*, *d* from 0.20 to 0.49); moderate (*f* from 0.25 to 0.40; *g*, *η*, *d* from 0.50 to 0.79); large (*f* > 0.40; *g*, *η*, *d* > 0.80).

## 3. Results

Below, the results of the analyses are shown extensively in the following order: preliminary analysis, parallelism, level equality, and flatness. Lastly, a section summarizing the results is given.

### 3.1. Preliminary Analyses

First, univariate normality was assessed. As reported in Table 1, the raw score of each dependent variable was normally distributed.

Second, multivariate normality was evaluated by means of Mardia’s multivariate kurtosis that was not statistically significant (*b*^2^*_p_* = 0.455, *p* = 0.649), suggesting the achievement of multivariate normality.

Third, the linearity of bivariate relationships among dependent variables was observed by means of a scatter matrix that revealed no curvilinear relationships. Multicollinearity among dependent variables was assessed using Pearson’s bivariate correlation coefficients that revealed the absence of multicollinearity (Table 2).

Finally, the homogeneity of the variance-covariance matrices was tested using Box’s *M* test, which was statistically significant (*M* = 132.103, *F* = 1.670, *p* < 0.001). However, according to the guidelines, Box’s *M* test could be overpowered when the groups have the same sample size—as in this case—and PA is quite robust to violations of this assumption [45,64]. Thus, considering these results, the PA was performed [45].

### 3.2. Profile Analysis (PA): Parallelism

The general null hypothesis of parallelism was rejected. A statistically significant interaction effect was found between the group of women (*IVO* vs. *CG* vs. *IVV*) and psychological variables related to violence, showing an absence of parallelism between profiles: Wilks’ Λ = 0.525, *F* = 6.615, *p* < 0.001, Cohen’s *f* = 0.616. This result revealed that the segments were different between profiles.

**Figure 1:**
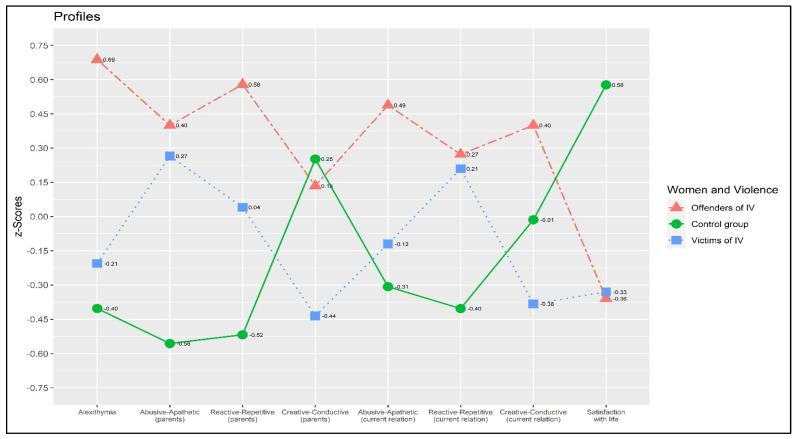
Plot of the profile analysis.

Figure 1 graphically represents the absence of parallelism.

### 3.3. Profile Analysis (PA): Level Equality—Between-Group Differences

Moreover, the general null hypothesis of level equality was rejected also. A statistically significant effect was found between the groups: *F* = 35.633, *p* < 0.001, Cohen’s *f* = 0.747. This result confirmed—once more—that the three groups were overall different, on average.

**Figure 2:**
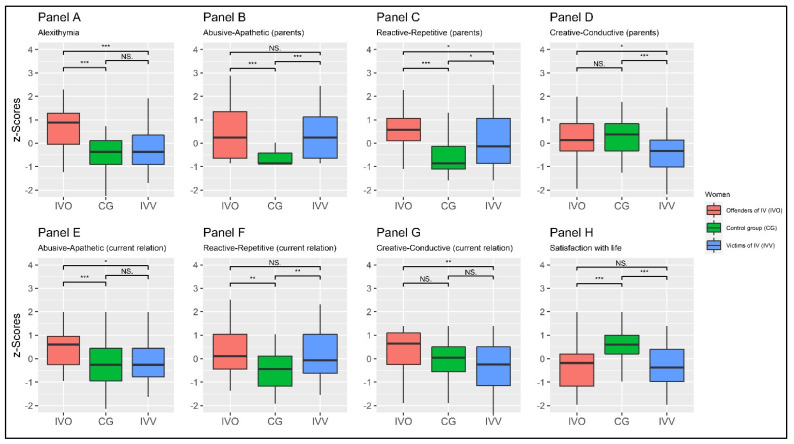
Plot of the between-group differences (level equality). Note: NS: *p* > 0.50; * *p* < 0.050; ** *p* < 0.010; *** *p* < 0.001;.

The multivariate pairwise-focused contrast between *IVO* and *CG* showed a statistically significant multivariate effect: Wilks’s Λ = 0.478, *F* = 16.492, and *p* < 0.001, *f* = 1.045. Moreover, the multivariate pairwise-focused contrast between *CG* and *IVV* showed statistically a significant multivariate effect: Wilks’s Λ = 0.780, *F* = 4.270, and *p* < 0.001, *f* = 0.531. Finally, a multivariate pairwise-focused contrast between *IVO* and *IVV* showed a statistically significant multivariate effect: Wilks’s Λ = 0.570, *F* = 11.394, and *p* < 0.001, *f* = 0.868. The results are summarized in Table 2 and Figure 2.

Taking into account TAS-20, MANOVA revealed statistically significant differences between the three groups: *F* = 18.477, *p* < 0.001, and *f* = 0.204. Furthermore, the univariate-focused contrast between the *IVO* and the *CG*’s means showed a statistically significant difference: *t* = 6.096, *p* < 0.001, *g* = |1.29|, *η* = 0.385. At the same time, the univariate-focused contrast between the means of the *CG* and the *IVV* revealed a non-statistically significant difference: *t* = −1.027, *p* = 0.562 *ns*, *g* = |0.22|, *η* = 0.759. Finally, the univariate-focused contrast between the means of *IVO* and *IVV* showed a statistically significant difference: *t* = 4.316, *p* < 0.001, *g* = |0.94|, *η* = 0.476. Results are reported in Table 2 and in Figure 2—panel A.

Taking into account the ‘pAA’ scale, MANOVA revealed statistically significant differences between the three groups: *F* = 14.956, *p* < 0.001, and *f* = 0.423. Furthermore, the univariate-focused contrast between means of *IVO* and the *CG* showed a statistically significant difference: *t* = 4.967, *p* < 0.001, *g* = |1.10|, *η* = 0.323. At the same time, the univariate-focused contrast between means of the *CG* and *IVV* revealed a statistically significant difference: *t* = −4.684, *p* < 0.001, *g* = |1.03|, *η* = 0.340. Finally, the univariate-focused contrast between means of *IVO* and *IVV* showed a non-statistically significant difference: *t* = 0.575, *p* = 0.502 *ns*, *g* = |0.13|, *η* = 0.834. Results are reported in Table 2 and in Figure 2—panel B.

Taking into account the ‘pRR’ scale, MANOVA revealed statistically significant differences between the three groups: *F* = 16.762, *p* < 0.001, and *f* = 0.512. Furthermore, the univariate-focused contrast between the means of the *IVO* and the *CG* showed a statistically significant difference: *t* = 6.647, *p* < 0.001, *g* = |1.41|, *η* = 0.305. At the same time, the univariate-focused contrast between the means of the *CG* and the *IVV* revealed a statistically significant difference: *t* = −2.736, *p* = 0.021, *g* = |0.60|, *η* = 0.574. Finally, the univariate-focused contrast between the means of *IVO* and *IVV* showed a statistically significant difference: *t* = 2.479, *p* = 0.041, *g* = |0.54|, *η* = 0.524. Results are reported in Table 2 and in Figure 2—panel C.

Taking into account the ‘pCC’ scale, MANOVA revealed statistically significant differences between the three groups: *F* = 6.296, *p* = 0.002, and *f* = 0.315. Furthermore, the univariate-focused contrast between the means of *IVO* and *CG* showed a non-statistically significant difference: *t* = −0.557, *p* = 0.843 *ns*, *g* = |0.12|, *η* = 0.716. At the same time, the univariate-focused contrast between the means of *CG* and *IVV* revealed a statistically significant difference: *t* = 3.644, *p* = 0.001, *g* = |0.77|, *η* = 0.546. Finally, the univariate-focused contrast between the means of *IVO* and *IVV* showed a statistically significant difference: *t* = 2.530, *p* = 0.035, *g* = |0.55|, *η* = 0.654. The results are reported in Table 2 and in Figure 2—panel D.

Taking into account the ‘cAA’ scale, MANOVA revealed statistically significant differences between the three groups: *F* = 8.348, *p* < 0.001, and *f* = 0.361. Furthermore, the univariate-focused contrast between the means of the *IVO* and the *CG* showed a statistically significant difference: *t* = 4.154, *p* < 0.001, *g* = |0.87|, *η* = 0.562. At the same time, the univariate-focused contrast between the means of the *CG* and the *IVV* revealed a *non*-statistically significant difference: *t* = −0.899, *p* = 0.642 *ns*, *g* = |0.19|, *η* = 0.757. Finally, the univariate-focused contrast between the means of *IVO* and *IVV* showed a statistically significant difference: *t* = 2.860, *p* = 0.015, *g* = |0.63|, *η* = 0.577. Results are reported in Table 2 and in Figure 2—panel E.

Taking into account the ‘cRR’ scale, MANOVA revealed statistically significant differences between the three groups: *F* = 7.006, *p* = 0.001, and *f* = 0.331. Furthermore, the univariate-focused contrast between the means of the *IVO* and the *CG* showed a statistically significant difference: *t* = 3.448, *p* = 0.003, *g* = |0.74, *η* = 0.597. At the same time, the univariate-focused contrast between the means of *CG* and the *IVV* revealed a statistically significant difference: *t* = −3.080, *p* = 0.008, *g* = |0.67|, *η* = 0.596. Finally, the univariate-focused contrast between the means of *IVO* and *IVV* showed a non-statistically significant difference: *t* = 0.269, *p* = 0.961 *ns*, *g* = |0.06|, *η* = 0.814. The results are reported in Table 2 and in Figure 2—panel F.

Taking into account the ‘cCC’ scale, MANOVA revealed statistically significant differences between the three groups: *F* = 6.855, *p* = 0.001, and *f* = 0.328. Furthermore, the univariate-focused contrast between the means of *IVO* and *CG* showed a non-statistically significant difference: *t* = 2.189, *p* = 0.079 *ns*, *g* = |0.47|, *η* = 0. 541. At the same time, the univariate-focused contrast between the means of *CG* and *IVV* revealed a non-statistically significant difference: *t* = 1.774, *p* = 0.186 *ns*, *g* = |0.38|, *η* = 0.701. Finally, the univariate-focused contrast between the means of *IVO* and *IVV* showed a non-statistically significant difference: *t* = 3.404, *p* = 0.003, *g* = |0.74|, *η* = 0.521. The results are reported in Table 2 and in Figure 2—panel G.

Taking SWLS into account, MANOVA revealed statistically significant differences between the three groups: *F* = 16.096, *p* < 0.001, and *f* = 0.502. Furthermore, the univariate-focused contrast between the means of *IVO* and the *CG* showed a statistically significant difference: *t* = −4.873, *p* < 0.001, *g* = |1.04|, *η* = 0.399. At the same time, the univariate-focused contrast between the means of the *CG* and *IVV* revealed a statistically significant difference: *t* = 4.965, *p* < 0.001, *g* = |1.06|, *η* = 0.391. Finally, the univariate-focused contrast between the means of *IVO* and *IVV* showed a non-statistically significant difference: *t* = −0.135, *p* = 0.990 *ns*, *g* = |0.03|, *η* = 0.776. The results are reported in Table 2 and in Figure 2—panel H.

### 3.4. Profile Analysis (PA): Flatness—Within-Group Differences

Finally, the general null hypothesis of flatness was not rejected. A non-statistically significant effect within groups was found: *F* = 0.063, *p* = 1, Cohen’s *f* = 0.063. This result suggested that there were no overall differences in the mean values of the dependent variables.

**Figure 3:**
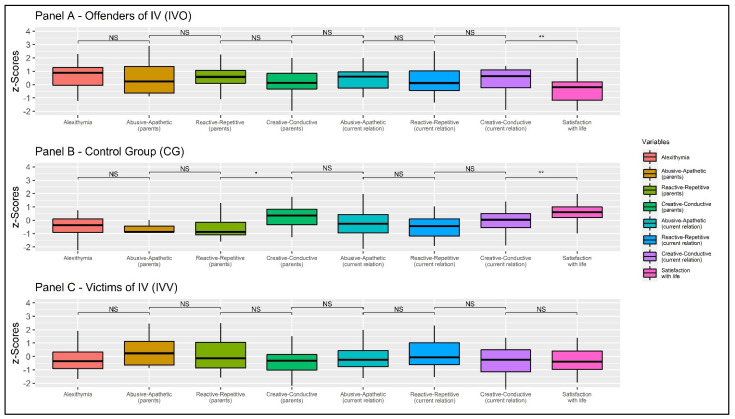
Plot of the within-group differences (flatness). Note: NS: *p* > 0.50; * *p* < 0.050; ** *p* < 0.010;.

Multivariate-focused contrast within *IVO* showed a statistically significant multivariate effect: Wilks’s Λ = 0.491, *F* = 5.029, and *p* = 0.001, *f* = 1.018. Furthermore, the multivariate-focused contrast within *CG* showed a statistically significant multivariate effect: Wilks’s Λ = 0.397, *F* = 9.121, and *p* < 0.001, *f* = 1.232. Finally, the multivariate-focused contrast within *IVV* showed a non-statistically significant multivariate effect: Wilks’s Λ = 0.703, *F* = 2.053, and *p* = 0.077, *f* = 0.650. The results are summarized in Table 3 and Figure 3.

Taking into account female *IVO*, the repeated measure ANOVA showed a statistically significant within subject effect: *F* = 4.598, *p* = 0.002, Cohen’s *f* = 0.339 (Greenhouse-Geissier correction). Focused contrasts (with Bonferroni correction) revealed a non-statistically significant difference within the first segment (TAS-20 vs. ‘pAA’ scale): *t* = 1.425, *p* = 1.000 *ns*, *d* = 0.223. Moreover, a non-statistically significant difference was found within the second segment (‘pAA’ scale vs. ‘pRR’ scale): *t* = −0.954, *p* = 1.000 *ns*, *d* = 0.149. A non-statistically significant difference was found within the third segment (‘pRR’ scale vs. ‘pCC’ scale): *t* = 1.768, *p* = 1000 *ns*, *d* = 0.276. A non-statistically significant difference was found within the fourth segment (‘pCC’ scale vs. ‘cAA’ scale): *t* = −1.555, *p* = 1.000 *ns*, *d* = 0.243. Furthermore, a non-statistically significant difference was found within the fifth segment (‘cAA’ scale vs. ‘cRR’ scale): *t* = 1.448, *p* = 1.000 *ns*, *d* = 0.226. In addition, a non-statistically significant difference was found within the sixth segment (‘cRR’ scale vs. ‘cCC’ scale): *t* = −0.546, *p* = 1.000 *ns*, *d* = 0.085. Finally, a statistically significant difference was found within the seventh segment (‘cCC’ scale vs. SWLS): *t* = 4.438, *p* = 0.002, *d* = 0.693. The results are reported in Table 3 (first row) and Figure 3 (Panel A).

Considering women of the *CG*, the repeated measure ANOVA showed a statistically significant within-subject effect: *F* = 12.716, *p* < 0.001, Cohen’s *f* = 0.514 (Greenhouse-Geissier correction). Focused contrasts (with Bonferroni correction) revealed a non-statistically significant difference within the first segment (TAS-20 vs. ‘pAA’ scale): *t* = 1.087, *p* = 1.000 *ns*, *d* = 0.155. Moreover, a non-statistically significant difference was found within the second segment (‘pAA’ scale vs. ‘pRR’ scale): *t* = −0.472, *p* = 1.000 *ns*, *d =* 0.067. A statistically significant difference was found within the third segment (‘pRR’ scale vs. ‘pCC’ scale): *t* = −3.752, *p* = 0.013, *d =* 0.536. Additionally, a non-statistically significant difference was found within the fourth segment (‘pCC’ scale vs. ‘cAA’ scale): *t* = 2.792, *p* = 0.210 *ns*, *d =* 0.399. Furthermore, a non-statistically significant difference was found within the fifth segment (‘cAA’ scale vs. ‘cRR’ scale): *t* = 0.926, *p* = 1000 *ns*, *d =* 0.132. In addition, a non-statistically significant difference was found within the sixth segment (‘cRR’ scale vs. ‘cCC’ scale): *t* = −2.045, *p* = 1.000 *ns*, *d =* 0.292. Finally, a statistically significant difference was found within the seventh segment (‘cCC’ scale vs. SWLS): *t* = −4.187, *p* = 0.003, *d =* 0.598. The results are reported in Table 3 (second row) and Figure 3 (panel B).

Considering female *IVV*, the repeated measure ANOVA showed a non-statistically significant within subject effect: *F* = 0.2.661, *p* = 0.052, Cohen’s *f* = 0.257 (Greenhouse-Geissier correction). Focused contrasts (with Bonferroni correction) revealed a non-statistically significant difference within the first segment (TAS-20 vs. ‘pAA’ scale): *t* = −2.402, *p* = 0.589 *ns*, *d =* 0.375. Moreover, a non-statistically significant difference was found within the second segment (‘pAA’ scale vs. ‘pRR’ scale): *t* = 1.593, *p* = 1.000 *ns*, *d =* 0.249. A non-statistically significant difference was found within the third segment (‘pRR’ scale vs. ‘pCC’ scale): *t* = 1.624, *p* = 1000 *ns*, *d =* 0.254. Additionally, a non-statistically significant difference was found within the fourth segment (‘pCC’ scale vs. ‘cAA’ scale): *t* = −1.273, *p* = 1.000 *ns*, *d =* 0.199. A non-statistically significant difference was found within the fifth segment (‘cAA’ scale vs. ‘cRR’ scale): *t* = −2.395, *p* = 0.598 *ns*, *d =* 0.374. In addition, a non-statistically significant difference was found within the sixth segment (‘cRR’ scale vs. ‘cCC’ scale): *t* = 2.019, *p* = 1.000 *ns*, *d =* 0.315. Finally, a non-statistically significant difference was found within the seventh segment (‘cCC’ scale vs. SWLS): *t* = −0.307, *p* = 1.000 *ns*, *d =* 0.048. The results are reported in Table 3 (third row) and Figure 3 (Panel C).

### 3.5. Summary of the Results

A profile per group has resulted from this study. The general null hypotheses of parallelism and level equality were rejected; therefore, a statistically significant difference between groups emerged from the MANOVA.

Specifically, the *IVO* group was statistically significant different from *CG* and *IVV* with respect to alexithymic impairments. The latter two groups were not significantly different. Regarding the relational styles experienced with parents, *IVO* and *IVV* differ significantly from *CG* in terms of the ‘pAA’ scale; however, there were no statistically significant differences between them. On the contrary, taking into account current relationships (‘cAA’ scale), the *IVO* group was statistically significant different from both *IVV* and *CG*, which, in contrast, did not show statistically significant differences. Regarding the ‘pRR’ scale, the three groups were statistically significant different. Regarding current relationships (‘cRR’ scale), the *CG* resulted in being statistically significant different from both *IVV* and *IVO*, which, in contrast, did not show statistically significant differences. Regarding the ‘pCC’ scale, *IVO* and *CG* were not statistically significant different. In contrast, *CG* was statistically significant different from the *IVV* group, which resulted in it being different from the *IVO* group as well. No statistically significant differences were found between the three groups on current relationships (‘cCC’ scale). Taking into account SWLS, *IVO* and *IVV* were not statistically significant different, but both differ significantly from the *CG*.

Moreover, the general null hypothesis of flatness was not rejected: the mean values of the dependent variables did not show overall differences.

## 4. Discussion

To date, IV represents a widespread phenomenon in modern society [1], but only a few studies have compared and profiled both *IVV* and *IVO*.

Indeed, both *IVV* and *IVO* may have had to deal with problematic parenting relationships (e.g., violent, neglecting). These experiences could have affected in an intergenerational way their style of interaction with current partners in an intergenerational way [31,33,34,35,66], hence influencing their SWL [67]. Moreover, they both show alexithymic impairments with a resulting difficulty identifying and understanding emotions [20,23,26].

Thus, this study aimed to profile and compare both sides of the coin of violence, victims, and perpetrators, by analyzing the aforementioned important variables related to IV: alexithymia, relational competence and styles, and SWL. Based on the results, the present study showed that there are statistically significant differences between *CG* and female *IVV* and *IVO*. First, according to several studies, both victims and offenders showed low SWL for the following different reasons [13,14]. On the one hand, female *IVV*’s low SWL may be due to the traumatic episodes of violence experienced [8]; on the other hand, the penitentiary regime where the female *IVO* lived may have contributed to their perception of low SWL [14]. Second, the group of women who exerted violence showed statistically significant higher levels of alexithymia than the *IVV* group and the *CG*, suggesting that *IVV* are almost comparable to the general population. These results are in line with the literature reporting that samples of violent offenders show difficulties in recognizing and understanding others’ emotions, including potential victims, revealing another element that could contribute to understanding violent offenders’ bursts of violence [21,68]. Finally, female *IVV*, as well as *IVO*, showed higher levels of dysfunctional relational styles in both past and current relationships [51] than the *CG*. Therefore, this result may suggest that people who had previous experiences with dysfunctional relational styles, including IV, can use similar styles in current relationships [68,69,70]—although in different ways.

### 4.1. Psychological Profiles

The psychological profiles delineated in this study deserve particular attention. Taking into account the CG: these women did not show difficulties related to expression as well as the comprehension of emotions. Furthermore, CC was the relational style experienced with parents and reproposed by them in current relationships. These results suggest that these women were able to interact with others functionally and communicate with their authentic self [38,51]. In summary, *CG* women reported being fully satisfied with their lives, being able to identify and manage their emotions and those of others, and seemed to be able to establish intimate relationships with others.

The profile emerged from *IVV* women was characterized by levels of alexithymia that were almost comparable, slightly higher but not statistically significant different from *CG*, revealing that *IVV* may not have difficulty identifying and labelling emotions [17]. Regarding relational competence and styles, *IVV* showed dysfunctional parental relationships and dysfunctional adult relationships. On the one hand, the most reported parental relational style was the AA one. This result suggests that *IVV* may have grown up in violent and neglecting contexts: indeed, they may have experienced parental violence, abuses, apathy, and neglect in previous relationships [36,37,68,71,72]. On the other hand, the current relational style reported the most was the RR one, revealing that *IVV* can react in a delayed manner to traumatic events. This result may represent a dysfunctional coping strategy with the possible consequent result of not modifying the interpersonal dynamics experienced [36,37]. In summary, female *IVV* are not satisfied with their lives; they seem to be able to identify their emotions and those of others. They probably grew up in a dysfunctional parental relational environment that may have influenced their deferred and tardive way of reacting to current painful relational events. Finally, the profile that emerged from *IVO* women showed the strongest statistically significant difficulties in identifying and labeling emotions. According to previous studies, alexithymic traits can affect these women’s ability to empathise with victims’ feelings and understand their emotions [23]. These alexithymic impairments can contribute to the increase in IVO’s behavioural expression, through bursts of violence [21]. Regarding relational competence and styles, *IVO* women reported dysfunctional relational styles in both previous and current relationships. On the one hand, they reported higher levels of parental relational styles of RR (slightly higher) and AA; thus, violence and apathy may characterize their childhood family environment [37,51]. On the other hand, they reported higher levels of both AA (slightly higher) and RR in current relationships, revealing that *IVO* can deal in a dysfunctional way with interpersonal exchanges [36,51]. In addition, this group of women also presented an unexpectedly high level of CC style, which may be due to the effect of the educational and rehabilitative nature of the penitentiary. In summary, *IVO* reported that they were unhappy with their lives; they showed high levels of alexithymia that hinder their recognition of their own and others’ emotions. Moreover, they probably grew up in a highly dysfunctional parental relational environment that presumably contributed to their way of reacting with violence to current relational events [14]. The profile depicted here resembles the symptomatology of antisocial personality disorder. Indeed, considering this study’s results, it is possible to assume that in both cases there seems to be no concern for others’ feelings or needs. In addition, there may be an inability to establish intimate relationships and a tendency to take dangerous actions without worrying about the consequences [73,74]. However, to our knowledge, there is a lack of research that investigates and proves the similarities and differences between antisocial personality disorder and *IVO* women with higher levels of the AA style.

The results of this study suggest that the relational competence and styles in each of the three groups of women in current relationships appear to be related to those experienced with caregivers in childhood [32,33,34,35,66]. Therefore, according to an intergenerational transmission perspective, these results may suggest that relational competence and styles seem to be passed down from caregivers to offspring [51,66,75,76]. It is important to highlight that the intergenerational transmission of relational competence and styles does not imply a causal relationship between past and present experiences [77]. Indeed, childhood interpersonal dynamics may represent one of the factors that may influence adult ways of interacting with others [77,78].

### 4.2. Limitations and Future Studies

However, despite the promising findings, this research presents some limitations. First, the self-report measures used may have been affected by the socially desirable response tendency. Second, although this study was based on a solid literature background, the research design was cross-sectional. In this way, it was not possible to fully verify the intergenerational transmission of relational competence and styles. Thus, future studies should fill this gap by conducting longitudinal studies aimed at exploring these constructs over time. Moreover, future research should evaluate the profiles here resulting. Furthermore, future studies should investigate the possible relationship between dysfunctional relational styles and antisocial personality disorder only mentioned here. In addition, future studies, as well as clinical interventions, should strongly consider that relational competence and styles could be transmitted from one generation to another one [32,33,34,35,51,66,79,80].

### 4.3. Clinical Implications

Considering the profiles outlined above, clinical interventions for both female *IVV* and *IVO* should focus on the empowerment of functional relational style (i.e., CC). Indeed, all of these women may be used to violent interpersonal dynamics from childhood to current relationships [33]. Therefore, with both female *IVO* and *IVV*, clinicians must develop psychological interventions aimed at improving a functional relational style, giving them the opportunity both to experience a new way of being with the other and to develop functional strategies for establishing interpersonal relationships. One way to achieve this goal could be by creating a strong therapeutic alliance that may represent a testing ground for subsequent relationships [79]. Another strategy would be to work with early benevolent memories to counterbalance negative relational exchanges of the past [80,81]. Additionally, psychological interventions on relational competence and styles include exercises and homework assignments [51,82]. One example is an exercise that can be used with couples and family members and aims to increase the level of intimacy. This exercise consists of sitting one in front of the other holding each other’s hands. Patients should focus on their “hurts” and express what they feel by loudly repeating “I hurt, I am hurting”. If patients manage to, they can articulate this sentence and they can also report how the sentences of others make them feel [82].

Considering *IVV*, although alexithymic impairments were almost equal to *CG*, this construct should be investigated in the clinical setting since according to the literature [26], women may develop alexithymic difficulties as a consequence of their trauma [20]. Therefore, psychological interventions should aim to reduce alexithymic difficulties since they represent a key factor in ‘healthy’ relationships.

On the contrary, considering *IVO*, psychological intervention should aim at the development of strategies that allow *IVO* to identify and understand emotions and affective states. This construct is important to be targeted in the clinical setting, as it may be one of the risk factors in the recidivism cycle of *IVO* [21]. According to the literature, interventions that could be conducted in this regard include psychotherapeutic interventions (e.g., CBT, psychoanalysis, etc.) aimed at improving patients’ understanding of emotions and their related bodily components. Moreover, psychoeducation involving skills training in emotion identification and regulation has shown to be effective with alexithymic patients [83]. In addition, group therapy can be used as it provides patients with the ability to observe each other identifying and describing their feelings, and they can improve this skill by receiving external feedback from group members [83].

Finally, it should be important to plan psychological interventions [54,84] aimed at improving SWL by promoting positive experiences.

## 5. Conclusions

IV represents a social emergency in the present society with negative psychological implications for the people involved [1,2,3]. Despite that, only a few studies deepened both sides of the coin of IV by comparing female *IVV* and female *IVO*. This research aimed to contribute to filling this gap in the literature by comparing these populations on three constructs that have not been fully investigated so far: alexithymia, relational competence and styles, and SWL. A profile per group of women has resulted from the study. On the one hand, *IVO* are the ones who display the strongest difficulties identifying and labelling emotions. Furthermore, they report having experienced dysfunctional relational styles in previous relationships, and this may be reflected in the relational styles currently present in their adult relationships. Indeed, all three kinds of relational styles are present in their current interpersonal exchanges; however, the most present seems to be the abusive-apathetic one. Both the *IVO* and *IVV* females seem to be strongly dissatisfied with their lives. Furthermore, the *IVV* group also reported having experienced dysfunctional relational styles in childhood. However, contrary to the *IVO* group, *IVV*’s most present relational style in current relationships seems to be the reactive-repetitive one. Furthermore, they do not seem to present alexithymic difficulties. These profiles have displayed similarities and differences between these populations on which it is possible to develop research considerations and to design psychological interventions. In conclusion, these findings suggest the planning of psychological interventions that should focus on the construction of new hopes, aims, and meanings of life.

## Figures and Tables

**Table 1:** Descriptive statistics of questionnaires (row scores) and correlations between variables.

		Descriptive Statistics				Correlations						
		*Mean*	*SD*	Sk	K	1	2	3	4	5	6	7
1	TAS-20	48.66	12.763	0.257	−0.639	-						
2	pAA	10.92	4.519	1.021	−0.025	0.267 ***	-					
3	pRR	13.59	4.166	0.413	−0.889	0.333 ***	0.580 ***	-				
4	pCC	19.42	4.322	−0.217	−0.308	−0.151 ^‡^	−0.577 ***	−0.502 ***	-			
5	cAA	21.48	5.810	0.303	−0.158	0.475 ***	0.234 **	0.335 ***	−0.172 *	-		
6	cRR	18.40	5.434	0.390	−0.499	0.373 ***	0.385 ***	0.508 ***	−0.283 **	0.617 ***	-	
7	cCC	30.69	6.699	−0.532	−0.350	−0.198 *	−0.318 ***	−0.131 ^‡^	0.557 ***	−0.278 ***	−0.301 ***	-
8	SWLS	14.95	5.075	−0.106	−0.774	−0.321 ***	−0.415 ***	−0.405 ***	0.389 ***	−0.428 ***	−0.479 ***	0.294 **

Note: *** *p* < 0.001; ** *p* < 0.010; * *p* < 0.050; ^‡^ *p* > 0.050 *ns.*; *Mean* = variable mean (raw score); *SD* = Standard deviation Sk = skewness; K = Kurtosis. TAS-20 = Toronto Alexithymia Scale 20; pAA = parental relational style Abusive-Apathetic; pRR = parental relational style Reactive-Repetitive; pCC = parental relational style Creative-Conductive; cAA = current relational style Abusive-Apathetic; cRR = current relational style Reactive-Repetitive; cCC = current relational style Creative-Conductive; SWLS = satisfaction with life scale.

**Table 2:** Between-group means comparison. Values are expressed as z-scores.

	*IVO*		*CG*		*IVV*		*IVO* vs. *CG*		*CG* vs. *IVV*		*IVO* vs. *IVV*	
	*Mean*	*SD*	*Mean*	*SD*	*Mean*	*SD*	*t*	*|g|*	*t*	*|g|*	*t*	*|g|*
TAS20	0.688	0.885	−0.403	0.796	−0.206	0.989	6.096 ***	1.29	−1.027 ^§^	0.22	4.316 ***	0.94
pAA	0.400	1.122	−0.557	0.560	0.265	1.000	4.967 ***	1.10	−4.684 ***	1.03	0.575 ^§^	0.13
pRR	0.579	0.819	−0.518	0.730	0.040	1.124	6.647 ***	1.41	−2.736 **	0.60	2.479 *	0.54
pCC	0.134	1.110	0.252	0.856	−0.436	0.921	−0.557 ^§^	0.12	3.644 ***	0.77	2.530 *	0.55
cAA	0.488	0.891	−0.308	0.921	−0.121	1.031	4.154 ***	0.87	−0.899 ^§^	0.19	2.860 **	0.63
cRR	0.273	1.047	−0.404	0.758	0.210	1.070	3.448 ***	0.74	−3.080 **	0.67	0.269 ^§^	0.06
cCC	0.400	0.966	−0.014	0.800	−0.383	1.112	2.189 ^§^	0.47	1.774 ^§^	0.38	3.404 **	0.74
SWLS	−0.360	1.001	0.578	0.784	−0.331	0.926	−4.873 ***	1.04	4.965 ***	1.06	−0.135 ^§^	0.03

Note: ^§^ *p* > 0.50 ns; * *p* < 0.050; ** *p* < 0.010; *** *p* < 0.001; *t* = *t*-test; *g* = Hedges’ *g* (effect size); *IVO* = offenders of interpersonal violence; *CG* = control group; *IVV* = victims of interpersonal violence; TAS-20 = Toronto Alexithymia Scale 20; pAA = parental relational style Abusive-Apathetic; pRR = parental relational style Reactive-Repetitive; pCC = parental relational style Creative-Conductive; cAA = current relational style Abusive-Apathetic; cRR = current relational style Reactive-Repetitive; cCC = current relational style Creative-Conductive; SWLS = Satisfaction with life scale.

**Table 3:** Within-group comparison.

	S1		S2		S3		S4		S5		S6		S7	
	TAS20 vs. pAA		pAA vs. pRR		pRR vs. pCC		pCC vs. cAA		cAA vs. cRR		cRR vs. cCC		cCC vs. SWLS	
	*t*	*|d|*	*t*	*|d|*	*t*	*|d|*	*t*	*|d|*	*t*	*|d|*	*t*	*|d|*	*t*	*|d|*
*IVO*	1.425 ^§^	0.223	−0.95 ^§^	0.149	1.768 ^§^	0.276	−1.555 ^§^	0.243	1.448 ^§^	0.226	−0.546 ^§^	0.085	4.438 **	0.693
*CG*	1.087 ^§^	0.155	−0.472 ^§^	0.067	−3.752 *	0.536	2.792 ^§^	0.399	0.926 ^§^	0.132	−2.045 ^§^	0.292	−4.187 **	0.598
*IVV*	−2.402 ^§^	0.375	1.593 ^§^	0.249	1.624 ^§^	0.254	−1.273 ^§^	0.199	−2.395 ^§^	0.374	2.019 ^§^	0.315	−0.306 ^§^	0.048

Note: ^§^ *p* > 0.50 ns; * *p* < 0.050; ** *p* < 0.010; S (…) = segment; *t* = *t*-test; *d* = Cohen’s *d* adapted formula for repeated measure comparisons (effect size); *IVO* = offenders of interpersonal violence; *CG* = control group; *IVV* = victims of interpersonal violence; TAS-20 = Toronto Alexithymia Scale 20; pAA = parental relational style Abusive-Apathetic; pRR = parental relational style Reactive-Repetitive; pCC = parental relational style Creative-Conductive; cAA = current relational style Abusive-Apathetic; cRR = current relational style Reactive-Repetitive; cCC = current relational style Creative-Conductive; SWLS = Satisfaction with life scale.

## Data Availability

The data presented in this study are available on request from the corresponding author. The data are not publicly available due to privacy restrictions.
